# Complete plastid genome sequence of the rare and endangered medicinal herb *Psammosilene tunicoides*, endemic to China

**DOI:** 10.1080/23802359.2019.1629348

**Published:** 2019-07-12

**Authors:** Jing Meng, Linna Zhang, Jun He, Yan Zhao

**Affiliations:** a College of Horticulture and Landscape, Yunnan Agricultural University, Kunming, China;; b Kunming Institute of Botany, Chinese Academy of Sciences, Kunming, China

**Keywords:** *Psammosilene tunicoides* W.C. Wu et C.Y. Wu, complete plastid genome, rare and endemic species, phylogenetic analysis

## Abstract

*Psammosilene tunicoides* W.C. Wu et C.Y. Wu, a monotypic species and endemic to Southwest China, is a rare and endangered traditional medicinal herb with satisfactory effects on multifold pathology. In order to provide crucial data for protection, we reported and analyzed the complete plastid genome of *P*. *tunicoides* as the foundation of germplasm conservation. The complete plastid genome is a typical quadripartite circular molecule of 153,957 bp in length, including a large single copy (LSC) region of 83,972 bp and a small single copy (SSC) region of 17,495 bp separated by two inverted repeat (IR) regions of 26,245 bp. In total of 111 unique genes were predicted, including 78 protein-coding genes (PCGs), 29 transfer RNA genes, and four ribosomal RNA genes. The overall GC content of *P*. *tunicoides* is 36.5%. The phylogenetic analysis revealed that *P*. *tunicoides* and *Dianthus caryophyllus* formed an independent clade with a 100% bootstrap support.

*Psammosilene tunicoides* W.C. Wu et C.Y. Wu belonging to *Psammosilene* W.C. Wu et C.Y. Wu, is a monotypic species and endemic to Southwest China, distributed only in Yunnan, Sichuan, Guizhou, and Tibet (Wu 1995). As a geoherb, *P. tunicoides* is one of the important ingredients of Chinese traditional medicine formulation ‘Yunnan Baiyao’ and has been listed in Chinese Pharmacopoeia, widely used for the treatment of fracture, rheumatic arthralgia, and hemorrhagic diseases (China Pharmacopeia Committee 2015; Yi et al. 2018). However, wild populations of *P. tunicoides* has been declined due to over-harvesting and habitat fragmentation, the species has become rare and endangered, thus has been listed as a Class II protected plant in China Plant Red Data Book (Fu 1992). In addition, due to the similar morphology between *Silene viscidula* and *P. tunicoides*, it is important to carry out identification and phylogenetic position studies of *P. tunicoides* (Kubitzki et al. 1993; Li et al. 2016; Wu 1995).

Fresh leaves of *P*. *tunicoides* were collected from the herbarium of Kunming Institute of Botany (KUN, Yunnan, China; 102°44′25.77″ E, 25°7′25.17″ N), and voucher specimens were also deposited at KUN. Total genomic DNA was extracted using modified CTAB method (Doyle and Doyle 1987). Reads of the plastid genome were assembled using CLC Genomic Workbench v10 (CLC Bio., Aarhus, Denmark). All the contigs were checked against the reference genome of *Agrostemma githago* (GenBank accession: KF527884) using BLAST (https://blast.ncbi.nlm.nih.gov/) and aligned contigs were oriented according to the reference genome. The complete plastid genomes were then constructed using Geneious v4.8.5 (Biomatters Ltd., Auckland, New Zealand) and were automatically annotated using DOGMA (http://dogma.ccbb.utexas.edu/). To identifying the phylogenetic position of *P*. *tunicoides*, 16 plastid genomes of Caryophyllaceae were aligned using the online program MAFFT (https://mafft.cbrc.jp/alignment/server/index/index.html), and the maximum likelihood (ML) tree was then conducted using MEGA v7.0 (Kumar 2016). The complete plastid genome was submitted to GenBank under the accession numbers of MK684403 for *P*. *tunicoides*.

The complete plastid genome of *P*. *tunicoides* represents a typical quadripartite circular molecule with 153,957 bp in length. It is composed by an LSC region of 83,972 bp, an SSC region of 17,495 bp and a pair of IR regions of 26,245 bp. In total of 111 unique genes were predicted, including 78 protein-coding genes (PCGs), 29 transfer RNA genes, and four ribosomal RNA genes. The overall GC content was 36.5%, the corresponding proportions of LSC, SSC, and IR regions were 34.2%, 30.0%, and 42.3%, respectively. Partial *rps19* and *ycf1* gene were annotated as pseudogene.

To investigate the phylogenetic position of *P*. *tunicoides*, 16 other complete plastid genomes from Caryophyllaceae were used to construct a phylogeny tree, using *Gymnocarpos przewalskii* (MF 795140) as the outgroups. The results showed that *P*. *tunicoides* clustered with *Dianthus caryophyllus* with 100% bootstrap support, indicated a closer relationship between the two species (Figure 1). The complete plastid genome of *P*. *tunicoides* will contribute to further phylogenetical studies and provide fundamental information for conservation strategies and molecular identification of this plant species.

**Figure 1.**
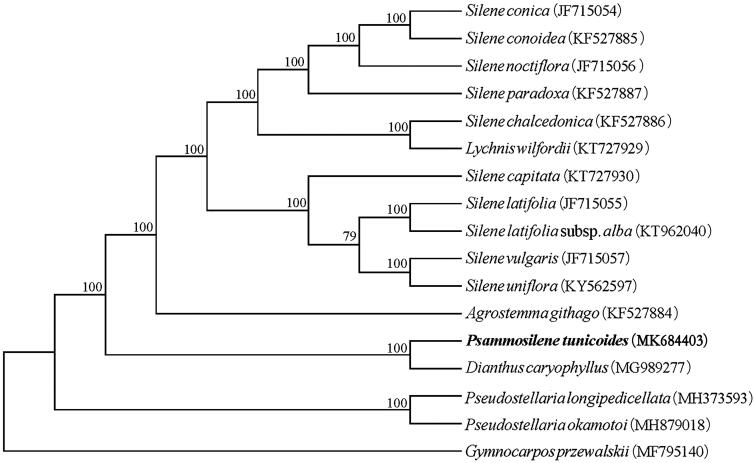
Phylogenetic relationship among 17 Caryophyllaceae species based on the maximum-likelihood (ML) analysis of the complete plastid genome sequences. Bootstrap support values (%) are indicated in each node.

## References

[CIT0001] China Pharmacopeia Committee 2015 Pharmacopeia of the people's republic of China (the first division). Beijing: Chinese Medical Science and Technology Press.

[CIT0002] Doyle JJ, Doyle JL 1987 A rapid DNA isolation procedure for small amounts of fresh leaf tissue. Phytochem Bull. 19:11–15.

[CIT0003] Fu LG 1992 China plant red data book: rare and endangered plants. Beijing: Science Press.

[CIT0004] Kubitzki K, Rohwer JG, Bittrich V 1993 The families and genera of vascular plants. Vol. 2. Flowering plants Dicotyledons: Magnoliid, Hamamelid and Caryophyllid families. Taxon. 43:517–518.

[CIT0005] Kumar S, Stecher G, Tamura K 2016 MEGA7: molecular evolutionary genetics analysis version 7.0 for bigger datasets. Mol Biol Evol. 33:1870–1874. 27004904 10.1093/molbev/msw054 PMC8210823

[CIT0006] Li J, Song M, Xiong C, Zhao B, Sun W 2016 Application of barcode high-resolution melting for rapid authentication of the medicinal plant *Psammosilene tunicoides*. Biotechnol Biotechnol Equip. 30:790–796.

[CIT0007] Wu ZY 1995 Flora Yunnan. Vol. 6 Beijing: Science Press.

[CIT0008] Yi C, Wu MX, Jie L, Ma XJ, Shi JL, Wang SN, Zheng ZQ, Guo JY 2018 Acute and sub-acute oral toxicity studies of the aqueous extract from radix, radix with cortex and cortex of *Psammosilene tunicoides* in mice and rats. J Ethnopharmacol. 213:199–209. 29137941 10.1016/j.jep.2017.11.011

